# Correspondence

**Published:** 1882-09

**Authors:** R. J. Walker

**Affiliations:** Scottsville, Ala.


### CORRESPONDENCE.

SCOTTSVILLE, ALA., Aug. 16th, 1882.

*Editor Atlanta Medical Register:*

Dear Sir—I notice that much is being said in the medical journals about the action of sierra salvia, or mountain sage, in fevers. It is claimed for it that it acts on the skin and kidneys. I think all the family of salvia would do the same. The object of this letter is, however, to bring to your notice another one of the salvias, or, what we call in this section, wild sage. It is used here a great deal in snake bites; the roots are beaten up and put into milk, the patient drinking of this freely; a poultice of it is also made and applied to the wound. It acts very promptly, giving relief almost instantly. I had one patient who was bitten by a ground rattle-snake, to whom it was administered. He was not affected by the bite at all; nothing else was given. Perhaps other practitioners have had some experience with the remedy, and, if so, I should be glad to hear what has been the result of their observations.

Respectfully, R. J. WALKER, M.D.

